# Supplementary material for: Increased processing of SINE B2 ncRNAs unveils a novel type of transcriptome deregulation in amyloid beta neuropathology
Source: eLife. 2020 Nov 16;9:e61265. doi: 10.7554/eLife.61265 (PMC7717908; doi:10.7554/eLife.61265)
Supplement: Transparent reporting form [file elife-61265-transrepform.docx]

***eLife’s* transparent reporting form**

We encourage authors to provide detailed information *within their submission* to facilitate the interpretation and replication of experiments. Authors can upload supporting documentation to indicate the use of appropriate reporting guidelines for health-related research (see [EQUATOR Network](http://www.equator-network.org/%20)), life science research (see the [BioSharing Information Resource](https://biosharing.org/)), or the [ARRIVE guidelines](http://www.plosbiology.org/article/info:doi/10.1371/journal.pbio.1000412) for reporting work involving animal research. Where applicable, authors should refer to any relevant reporting standards documents in this form.

If you have any questions, please consult our Journal Policies and/or contact us: [editorial@elifesciences.org](mailto:editorial@elifesciences.org).

**Sample-size estimation**

- You should state whether an appropriate sample size was computed when the study was being designed
- You should state the statistical method of sample size computation and any required assumptions
- If no explicit power analysis was used, you should describe how you decided what sample (replicate) size (number) to use

Please outline where this information can be found within the submission (e.g., sections or figure legends), or explain why this information doesn’t apply to your submission:

Sample size requirements were estimated based on previous results on application of the same type of short RNA sequencing in mouse (Zovoilis et al, Cell 2016). For similar sequencing depth and sequencing mode that study employed two generated datasets per condition, a number that was increased in the current study by one (3 datasets per age per condition, except 3m WT-see below), in total 17 datasets. Sample size for the long RNA seq experiments and confirmatory qPCR experiments followed that of short-RNA seq. This information can be found both in methods section under “Animals and behavioural measurement”and the legends of each figure as well as in the metadata of the GEO submitted experiment with access number GSE149243.

**Replicates**

- You should report how often each experiment was performed
- You should include a definition of biological versus technical replication
- The data obtained should be provided and sufficient information should be provided to indicate the number of independent biological and/or technical replicates
- If you encountered any outliers, you should describe how these were handled
- Criteria for exclusion/inclusion of data should be clearly stated
- High-throughput sequence data should be uploaded before submission, with a private link for reviewers provided (these are available from both GEO and ArrayExpress)

Please outline where this information can be found within the submission (e.g., sections or figure legends), or explain why this information doesn’t apply to your submission:

For every age group in each condition we used material from three different mice (biological replicates), with the exception of WT 3m old mice: During the hippocampal RNA extractions, the RNA of one of the three 3m old WT mice had very low RIN scores, which could be a confounding factor for identification of SINE RNA fragments by the short-RNA-seq. As this happened some months after the hippocampal extractions we did not have available other 3 month mice of the same cohort used in our previous study of these mice for the behavioral and IHC studies. Thus, in order to allow comparisons with the IHC and behavioral data we included only two replicates in this condition and did not sequence another WT 3m old mouse. This is mentioned in methods under section “Animals and behavioural measurement”. Number of mice in each group is mentioned in the legend of the respective figures. For cell culture experiments we used 4 biological replicates for the control peptide vs amyloid beta peptide (42) and 3 biological replicates for the treatment with anti-Hsf1. These numbers are mentioned in the figure legends. For confirmatory qPCR experiments we used three technical replicates for each biological replicate, and the same number of biological replicates corresponding to mice and cell culture experiments mentioned above.

**Statistical reporting**

- Statistical analysis methods should be described and justified
- Raw data should be presented in figures whenever informative to do so (typically when N per group is less than 10)
- For each experiment, you should identify the statistical tests used, exact values of N, definitions of center, methods of multiple test correction, and dispersion and precision measures (e.g., mean, median, SD, SEM, confidence intervals; and, for the major substantive results, a measure of effect size (e.g., Pearson's r, Cohen's d)
- Report exact p-values wherever possible alongside the summary statistics and 95% confidence intervals. These should be reported for all key questions and not only when the p-value is less than 0.05.

Please outline where this information can be found within the submission (e.g., sections or figure legends), or explain why this information doesn’t apply to your submission:

For long RNA seq, statistical analysis was performed based on the p-values calculated for each gene by DESeq2 (using p val < 0.05 as threshold) followed by correction for double testing and FDR calculation again by DESEq2. This is mentioned in methods section under “Bioinformatics analysis”. For functional annotation term enrichment analysis, EASE Score, a modified Fisher Exact p-value applied by the DAVID annotation platform was used (see methods) with an EASE score reporting threshold of 0.05. This is mentioned in methods section under “Bioinformatics analysis”. Black line in boxplots throughout the manuscript denote the median. Error bars in bar graphs represent standard deviation from the mean. P values for statistical significance in comparisons of gene expression levels or B2 RNA levels and processing based on RNA-seq or short-RNA-seq data were calculated using unpaired non-directional t-test, with p=0.05 as the threshold for statistical significance. When applicable, p-values of key questions above this threshold are also reported. P values for statistical significance in comparisons of qPCR data that are confirmatory to a specific difference observed in the RNA-seq data, were calculated using unpaired directional t-test, with p=0.05 as the threshold for statistical significance. N numbers, methods, statistical test used and significance thresholds are reported in the respective legend of each figure.

(For large datasets, or papers with a very large number of statistical tests, you may upload a single table file with tests, Ns, etc., with reference to sections in the manuscript.)

**Group allocation**

- Indicate how samples were allocated into experimental groups (in the case of clinical studies, please specify allocation to treatment method); if randomization was used, please also state if restricted randomization was applied
- Indicate if masking was used during group allocation, data collection and/or data analysis

Please outline where this information can be found within the submission (e.g., sections or figure legends), or explain why this information doesn’t apply to your submission:

Group allocation was done based on the genotype and age of each mouse. This is described in methods and in the experimental design of Fig.2.

**Additional data files (“source data”)**

- We encourage you to upload relevant additional data files, such as numerical data that are represented as a graph in a figure, or as a summary table
- Where provided, these should be in the most useful format, and they can be uploaded as “Source data” files linked to a main figure or table
- Include model definition files including the full list of parameters used
- Include code used for data analysis (e.g., R, MatLab)
- Avoid stating that data files are “available upon request”

Please indicate the figures or tables for which source data files have been provided:

Raw sequence source data are available through GEO with access number GSE149243. Source data for gene expression FPKM or B2 RNA fragments counts are available through GEO under files entitled “processed”. PAGE gels for the incubations have been uploaded as two source files for Fig.5. Source information for DEseq2 output for differential expression(including statistics and log fold change differences) is provided as suppl. tables. Full lists of all reported gene ontology terms are provided as supplementary tables 4 and 8. Source information for correlation co-efficient is provided as Suppl. Table 12. Figure Gene lists of B2 SRGs and randomly selected genes are provided in separate tables in supplemental data (Suppl. Table1 and 13, respectively). DESeq2, bwa, HISAT2, samtools are publicly available software packages.
